# Next Generation Sequencing of Genotype Variants and Genetic Association between Heat Shock Proteins HSPA1B Single Nucleotide Polymorphism at the g.31829044 Locus and Heat Tolerance: A Pilot Quasi-Experimental Study

**DOI:** 10.3390/biom12101465

**Published:** 2022-10-12

**Authors:** Faith O. Alele, John R. Otto, Bunmi S. Malau-Aduli, Aduli E. O. Malau-Aduli

**Affiliations:** 1 College of Healthcare Sciences, James Cook University, Townsville, QLD 4811, Australia; 2 College of Public Health, Medical and Veterinary Sciences, James Cook University, Townsville, QLD 4811, Australia; 3 College of Medicine and Dentistry, James Cook University, Townsville, QLD 4811, Australia

**Keywords:** heat tolerance, heat shock proteins, HSPA1B, HSP90AA2, exertional heat stroke, exertional heat illness, military, genetics, polymorphism

## Abstract

Heat tolerance and exertional heat stroke (EHS) are rare health conditions that have been described and characterised but have never been genetically solved. Knowledge of the role of single nucleotide polymorphisms (SNPs) in heat shock proteins (HSPs) genes and their associations with heat tolerance and EHS is limited. This pilot study aimed to identify SNP in HSPA1B, HSP90AA2 and DNAJA1 genes and their associations with heat tolerance and EHS history in a quasi-experimental design. Participants comprised Australian Defence Force members (ADF) who had a history of EHS and the general population. Genomic DNA samples were extracted from the venous blood samples of 48 participants, sequenced and analysed for SNP. Forty-four per cent (44%) of the participants were heat intolerant, and 29% had a history of EHS. Among participants with a history of EHS, there was an association between heat tolerance and HSPA1B SNP at the g.31829044 locus. However, there were no associations between HSPA1B and HSP90AA2 SNP and heat tolerance. All participants had the same distribution for the DNAJA1 SNP. In conclusion, the findings indicate an association between the HSPA1B genetic variant at the g.31829044 locus and heat tolerance among ADF participants with a history of EHS. Further research with a larger number of military participants will shed more light on the associations between HSP genes and heat tolerance.

## 1. Introduction

Heat shock proteins (HSPs) are molecular chaperones that are constitutively expressed under normal physiological conditions [1]. However, they may be upregulated or overexpressed in response to oxidative, physiological and environmental stresses [2,3]. HSPs are classified by molecular mass ranging from <27 to >110 kDa [4,5], and findings suggest that heat shock protein (HSP) genetic polymorphisms are associated with different diseases, including cardiac death, depression, age-related hearing impairment, glaucoma and infertility [6,7,8,9,10]. However, less is known about HSP genetic polymorphism, heat stress and exercise. Available evidence indicates that HSPs may be upregulated during exercise in physically active individuals, depending on the level of physical activity [11]. In addition, during exertion in hot environments, heat stress may occur with a quantifiable HSP gene expression [4]. There are suggestions that cellular mechanisms, such as modifications in HSP gene expression, may help individuals cope with thermoregulatory changes during heat stress [4]. However, it is unknown if genetic variations in heat shock proteins (HSP) play a role in heat tolerance.

Heat intolerance is known as the inability to withstand heat stress during exercise in hot environments [12]. In military populations such as the Australian Defence Force (ADF), heat intolerance increases the risk of exertional heat illness (EHI) with a higher risk of exertional heat stroke (EHS). EHI is a significant threat to military personnel during training and combat activities undertaken in hot conditions and may be associated with heat intolerance [13,14]. Lisman et al. [15] and Shapiro et al. [16] reported that approximately 28% and 100% of the US Armed Forces and Israeli Defence Force members, respectively, with a history of EHS were heat intolerant. In Australia, 56% of ADF members referred for heat tolerance testing following a known or suspected EHS event were reported to be heat intolerant [17]. Early studies identified dehydration, acclimatisation, obesity, low physical fitness, infectious disease, drugs and low work efficiency as predisposing factors for heat intolerance [12]. More recent studies have found aerobic fitness, age, gender, body fat per cent, creatinine, and cortisol, as physiological and biochemical predictors of heat intolerance [15,17]. Yet, the underlying genetic mechanism of heat intolerance is not fully understood because it is still unclear why some individuals cannot sustain heat stress under the same environmental conditions with comparable activity levels while others can [12].

To our current knowledge, no study has investigated the role of genetic polymorphisms in heat intolerance and exertional heat illness (EHI). Heat intolerance may reflect the genetic characteristics of the individual [18]. While it is evident that HSPs are modulated during heat illness [19,20,21], there is limited knowledge on genetic polymorphisms in heat intolerance. Evidence shows that when peripheral blood mononuclear cells obtained from healthy adults were exposed to heat shock at 43 °C significant changes in HSPs expression were observed [22], and the HSPs were significantly upregulated in participants with EHI history compared to controls [23]. Thus, it is crucial to identify genetic factors that may help to explain susceptibility to heat intolerance and serve as biomarkers for identifying those at risk of EHI. In this study, a targeted sequencing approach of *HSP70-2* or *HSP72 (HSPA1B)*, *HSP90AA2* (an isoform of HSP 90), and *DNAJA1*(*HSP 40A1*) was justifiable because *HSPA1B* has been previously shown to be a highly inducible isoform of *HSP72* and facilitates positive heat adaptations and cellular responses during exercise [24]. Similarly, HSP90 is induced and increases in response to exercise in the heat and core temperature elevation [24], while HSP40 stabilises and delivers client proteins required by HSP70 and HSP90 to perform their functions [25,26]. Besides, most of the current and previous HSP studies focused on messenger RNA and gene expression investigations with limited evidence on the identification of genetic polymorphisms and their associations with heat intolerance. Given that heat intolerance may be associated with career ramifications for military personnel, including the possibility of medical discharge [17], it is imperative to identify genetic polymorphisms associated with heat intolerance. In addition, HSPs influence heat stress and cytoprotection, hence the need to investigate *HSPA1B*, *HSP90AA2* and *DNAJA1* polymorphisms and their associations with heat intolerance [24].

Therefore, the primary aim of this study was to identify if differences exist in SNP in *HSPA1B*, *HSP90AA2* and *DNAJA1* genes associated with heat intolerance in Australian military populations with a history of EHS compared to the general population (controls). It was hypothesised that heat intolerant participants would exhibit differential genotypes for *HSPA1B*, *HSP90AA2* and *DNAJA1* SNP compared to heat tolerant participants with or without a history of EHS.

## 2. Materials and Methods

As part of a more extensive study investigating the predictors of heat intolerance in military populations, a quasi-experimental design was utilised in this study [17]. After an EHS event in the ADF, the return to duty process involves using the standard heat tolerance test (HTT) developed by the Israel Defence Force to determine heat tolerance status [14,16].

### 2.1. Participants

Participants were military personnel (*n* = 14), university staff, students and athletes (*n* = 34) between the ages of 18 and 60 years (to match the ADF’s demographic profile). Military personnel with a previous or suspected history of EHS referred for the HTT and volunteered to participate in the study were recruited. The other participants were athletes or staff and students of James Cook University and were recruited via email or verbal advertisement and provided with the information sheet about the study. Medical history was obtained via screening forms in person or by email. Written consent was obtained from all participants prior to participation in the study. Exclusion criteria for the study were: (1) A history of malignant hyperthermia, hypertension, or diabetes; (2) Undergoing treatment for a mental disorder; (3) Pregnant or lactating women; (4) Undergoing treatment for anaemia, and (5) Using prednisolone or beta-blockers or glucose-lowering agents. All participants were advised to avoid exhaustive exercise, alcohol, caffeine and heat exposure 24 h before taking part in the HTT. Full details of the methodology have been previously published [17].

### 2.2. Heat Tolerance Test Procedure

The HTT was conducted in the morning in a climate control chamber set to 40 °C and 40% relative humidity (hot/dry conditions). Before the test commenced, hydration status was assessed using urine specific gravity (USG) (Atago handheld refractometer, Atago Co., Ltd., Itabashi, Tokyo, Japan). If the USG was >1.015, rehydration commenced with water, and another assessment of the USG was performed. Resting heart rate (Polar T31 Coded Transmitter, Polar Electro Oy, Kempele, Finland) was measured before the test commenced. Similarly, blood pressure at rest (Aneroid Sphygmomanometer Two-Handed, ALP-K2, Tokyo, Japan) was also measured before the test started. During the HTT, participants walked on a treadmill for 2 h at 5 km/h with a 2% incline. Rectal core temperature (RET-1 Rectal Probe, Physitemp Instruments, LLC, Clifton, NJ, USA) and heart rate were measured at the start of the test and assessed every 5 min during the test. Participants were classified as heat intolerant if the core temperature was >38.5 ℃, the heart rate (HR) was >150 bpm, or when the core temperature failed to plateau (increase >0.45 °C during the 2nd hour of the HTT). The HTT was discontinued if the participants requested to stop the test or experienced any symptoms of heat illness (including nausea, headaches, weakness, dizziness, etc.). Participants had *ad libitum* access to water during the test.

### 2.3. Blood Collection and Deoxyribonucleic Acid (DNA) Extraction

Blood samples were collected from each of the 48 participants in 4mL ethylenediaminetetraacetic acid (EDTA) containing tubes and stored at −80 °C until needed for genomic DNA extraction. Genomic DNA was extracted from the whole blood samples using NucleoSPin Blood Kits per package protocol (Scientifix Pty Ltd., Clayton, VIC, Australia). DNA concentration was measured from the absorbance at 260 nm and 280 nm using a NanoDrop ND-1000 spectrophotometer (NanoDrop, Analytical Technologies, Biolab). The ratio of absorbance at 260/280 nm was used to quantify DNA purity.

### 2.4. Primer Design

HSP primers were designed using the Geneious Prime Software Program 2020 version.2.2, Auckland, New Zealand (http://www.geneious.com, accessed on 25 April 2020) and synthesised at Integrated DNA Technologies Pty. Ltd., Melbourne, Australia (itddna.com). Reference coding sequences of HSPA1B, DNAJA1 and HSP90AA2 genes were obtained from the National Center for Biotechnology Information (NCBI) database (Genbank). The forward and reverse primer sequences, fragment lengths and annealing temperatures are shown in Table S1.

### 2.5. Polymerase Chain Reaction (PCR)

Platinum™ SuperFi™ II PCR Master Mix (Thermofisher Scientific, Melbourne, Australia) was used for PCR amplification of the HSPA1B, DNAJA1 and HSP90AA2 genes. The PCR utilised 25 µL of 2× Platinum™ SuperFi™ II PCR Master Mix (Thermofisher Scientific, Melbourne, Australia), 0.5 µM of each primer (IDT, Australia), and 100 ng of the DNA template to make up a total volume of 50 µL in a The SimpliAmp™ Thermal Cycler (Thermofisher Scientific, Melbourne, Australia). The PCR conditions were: Initial denaturation for 1 min at 98 °C (1 cycle); denaturation for 15 s at 98 °C; annealing at 60 °C for 15 s and extension for 9 min at 72 °C. A final extension was performed at 72 °C for 9 min. After a total of 35 cycles, the PCR product was confirmed using 0.8% agarose gel electrophoresis.

### 2.6. PCR Clean-Up

The PCR products were cleaned using Sera-Mag™ SpeedBeads in a Zephyr NGS Workstation (Caliper Lifesciences, Perkin-Elmer, Waltham, MA, USA). The cleaned PCR products were quantified using a Promega dsDNA Quantifluor System Kit (Ref: E2670, 00002484139), Quantifluor dsDNA System (Promega, Madison, WI, USA) at 0.4 nM to ensure adequate coverage during sequencing. The products were normalised to 2 ng/µL and diluted to 0.2 ng/µL using 10 mM Tri-Hydrochloride (PH 8.0) in readiness for library preparation using the Illumina Nextera XT DNA.

### 2.7. Library Preparation, Quantification and Sequencing

Nextera XT DNA Library Prep kit (Illumina, CA, USA) was used to prepare the libraries following the manufacturer’s protocol. Only incorporated adapters were included, and the fragment size and concentration of each DNA library using Agilent High Sensitivity D5000 reagents and ScreenTape on the Tape Station 4200 Instrument (Agilent Technologies, Santa Clara, CA, USA). Concentration estimates of each DNA library were also obtained using QuantiFluor^®^ dsDNA System (Promega, Madison, WI, USA). The libraries were normalised to 4nM using 10 mM Tris-HCl (pH 8.5) before pooling. The obtained DNA libraries were sequenced on an Illumina MiSeq benchtop sequencer, using a 500-cycle MiSeq Reagent Nano Kit (Illumina, Inc., San Diego, CA, USA).

### 2.8. SNP Detection

The DNA sequence was analysed using Geneious Prime software program 2022.1.1 (http://www.geneious.com, accessed on 10 May 2022). Reference sequences in the NCBI for *HSPA1B* (NC_000006.12), *DNANJA1* (NC_000009.12), and *HSP90AA2* (NC_000011.10) were used for comparative analysis. The raw reads obtained after sequencing were assessed for quality using the Geneious Prime software. Low-quality reads and adapters were trimmed using BBDuk trimmer in Geneious Prime 2020 v.2.2. The SNP were called using the following parameters: Phred score quality (Q) value was set at 20 to increase the likelihood of calling true SNP to 99%. Short reads with a minimum length of 20 bp were discarded, and regions of low coverage were excluded. The reads were mapped to the reference sequences obtained from NCBI databases with sensitivity set on Medium Sensitivity/Fast and Fine-Tuning (iterate up to 5 times) option was selected to improve the results.

### 2.9. Statistical Analyses

All statistical analyses were performed using the Statistical Package for Social Sciences (SPSS Statistics, Version 26, IBM Corp, Armonk, New York, NY, USA) and R Project for Statistical Computing, Vienna, Austria (version 3.6.3). Following adjustments for sex, age and heat tolerance status, the genetic distribution of the major allele frequencies from the sequence data was analysed using the Hardy–Weinberg equilibrium principle described by Graffelman et al. [27]. Linkage disequilibrium as an index of non-random association between alleles of different loci, was estimated as the difference between the frequency of gametes carrying the pair of alleles A and B at two loci (pAB) and the product of the frequencies of those alleles (pA and pB), D_AB_ = pAB − pApB, where the allele pair AB is a haplotype and pAB is the haplotype frequency [28]. Heat tolerance status was analysed as a categorical dichotomous variable (heat tolerant versus heat intolerant) and also defined using final core temperature (Tc) and final heart rate (HR) as continuous variables. Chi-square (χ^2^) and Fisher’s exact tests were used to compare the frequency of the genotypes by heat tolerance status and EHS history. False discovery rate (FDR) was used to correct the *p*-values from the stratified analysis. The relationship between heat tolerance status and the genotype stratified by EHS was also assessed using Fisher’s exact test and Chi-Square tests. Residual correlations between HSP SNP, final core temperature and heart rate were estimated after independent samples *t*-test and One-way Analysis of variance (ANOVA) was used to identify the final Tc and HR differences between the HSP90AA2 and HSPA1B genotypes. 

## 3. Results

The mean age of the 48 participants was 32 ± 12 years. Males accounted for 71% (*n* = 34) of the participants, while 29% (*n* = 14) had a history of EHS. Overall, 44% (*n* = 21) of the participants were heat intolerant. Among those who were heat intolerant, 38.1% (*n* = 8) had a history of EHS, while 61.9% (*n* = 13) had no history of EHS (Table 1).

The allele distributions of the HSP SNP genotypes were in Hardy–Weinberg equilibrium and are shown in Table S2. The identified *HSPA1B* SNP was located at the g.31829044 and g.31829851G>C loci, while *HSP90AA2* SNP was located at the loci g.27889377T>A, g.27890100C>T and g.27890332T>C, with major allele frequencies ranging from 51–85%. *DNAJA1* SNP was the same for all the participants; hence, there was no basis for comparison, and the data was not tabulated.

### 3.1. Heat Tolerance Status and HSP SNP

As shown in Table 2, there were no significant differences between the *HSPA1B* SNP genotypes (χ^2^ = 1.431, df = 2, *p* = 0.489, and χ^2^ = 0.741, df = 2 *p* = 0.690) at all loci regardless of heat tolerance status. There was a similar pattern for the *HSP90AA2* SNP genotypes (*p* = 0.978).

### 3.2. HSP SNP Differences in Final Core Temperature and Heart Rate

**Figure 1:**
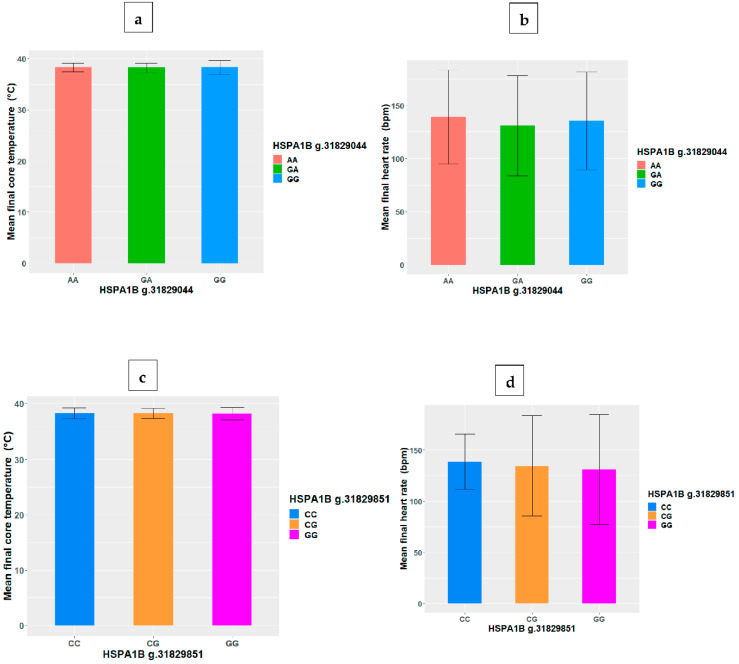
Variations in HSPA1B SNP genotype mean (+/−SD) core temperature and heart rate at loci (**a**,**b**) g.31829044 and (**c**,**d**) g.31829851.

**Figure 2:**
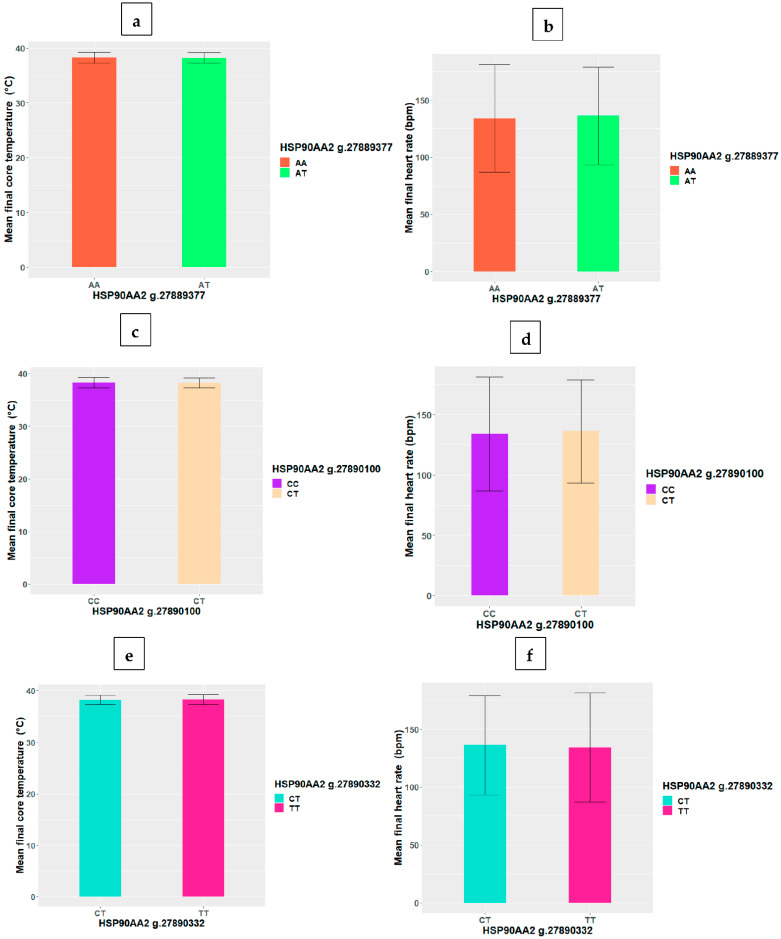
Variations in HSP90AA2 SNP genotype mean (+/−SD) core temperature and heart rate at loci (**a**,**b**) g.27889377, (**c**,**d**) g.27890100 and (**e**,**f**) g.27890322.

As depicted in Table 3, there were no significant correlations (*p* > 0.05) between the HSP SNP, final core temperature and heart rate. Similarly, Figure 1 and Figure 2 show that there were no significant differences (*p* > 0.05) between genotypes in final core temperature and heart rate at all the HSP SNP loci.

### 3.3. HSP SNP and EHS History

There was no significant difference in the *HSPA1B* SNP genotype between those who had a history of EHS (ADF members) and the controls (without a history of EHS). Similarly, the polymorphisms for *HSP90AA2* were not significantly different between those with or without the history of EHS (Table 4).

### 3.4. HSP SNP and Heat Tolerance Stratified by EHS History

Interestingly, after stratification by EHS history, there was a significant difference in the *HSPA1B* SNP at the g.31829044 locus between the heat tolerant and intolerant within the ADF group, i.e., those with a history of EHS (Fisher’s exact test = 6.969, df = 2, *p* = 0.0240) (Table 5). In the ADF group, 100% of the heat tolerant participants had the AG genotype at the g.31829044 locus compared to 25% of heat intolerant participants. FDR analysis verified the results (FDR-*p* = 0.048). There was no difference observed in the HSPA1B SNP genotype between the heat tolerant and intolerant control participants (without a history of EHS). There was no difference in the other *HSPA1B* SNP loci genotype between the heat tolerant and intolerant within each group (ADF and controls, respectively). Similarly, *HSP90AA2* polymorphism showed no significant difference with heat tolerance status within the groups (ADF and controls) (Table 5).

## 4. Discussion

During heat stress, changes in HSPs expression occur to activate a heat shock response [4]. Evidence suggests that HSP polymorphisms play a crucial role in disease manifestation by causing alterations in sensitivity to heat stress [29]. While changes in HSP expression (over and under expression) in military personnel with EHI have been reported, little is known about the role of underlying genetic polymorphisms in heat tolerance [23]. Given that the environment in which military personnel operate is dynamic and unpredictable, it is important to identify strategies to minimise the risk of EHI and heat intolerance [30]. Heat tolerance needs to be determined before returning to duty after an episode of EHS; therefore, identifying the SNP associated with heat intolerance is crucial.

This study investigated the association between *HSPA1B* and *HSP90AA2* SNP and heat intolerant versus heat tolerant individuals and found no significant differences. When heat tolerance status was stratified by EHS history, a significant difference between *HSPA1B* SNP at g.31829044 locus and heat tolerance in only ADF participants with a history of EHS was observed. All participants with an EHS history who were heat tolerant had the AG genotype at the *HSPA1B* SNP g.31829044 locus. This implies that the AG genotype may be a protective marker for heat stress. Given that there are different risk factors for EHS, other predisposing factors may have been involved in the initial heat stroke episode [31]. For example, motivation is considered a factor that increases the risk of EHS, wherein military personnel may over-exert themselves and not self-pace during activities in the heat [31]. Where self-pacing is compromised, even the protective role of the HSP will be over-ridden, and heat illness may occur [32].

The AG genotype is heterozygous, and genomic heterozygosity is demonstrated to have a significant role in promoting benefits for survival and reproduction [33]. This heterozygous advantage has been observed in animals and humans. For example, reduced heterozygosity in crows was associated with increased susceptibility to vector-borne diseases [34]. In humans, previous studies have shown that healthy ageing and human health span were associated with genomic heterozygosity [33]. Heterozygosity has been linked to better health-related characteristics, including reduced blood pressure and low-density lipoprotein (LDL) cholesterol [35]. Additionally, there have been indications of a strong correlation between genome-wide heterozygosity and the reduced risk of death [36]. However, given such a small group with an EHS history in the study, it cannot be completely ruled out that the study findings may be due to the larger number of carriers of the heterozygous genotype (AG).

It is also possible that the presence of the AG genotype may influence the expression of the *HSPA1B* gene. The precise mechanism by which the genotype influences the expression of *HSPA1B* was not studied; however, the homozygous genotype GG has been reported to be associated with reduced *HSPA1B* expression and was a negative predictor of survival in patients with small-cell lung cancer [37]. In addition, patients with small cell lung cancer with the AG genotype had a higher survival rate than those with the homozygous genotype -GG [37]. In developmental studies, it was found that in very low birth weight neonates, the *HSPA1B* GG genotype was associated with low HSP 72 (HSPA1B) expression and was also associated with the risk of premature birth and acute renal failure [24]. Studies have also shown that the AG genotype is associated with a lower risk of susceptibility to different diseases, including idiopathic pulmonary fibrosis, glaucoma, and noise-induced hearing loss [10,38]. *HSPA1B* polymorphisms have also been reported to be associated with coronary artery disease [39], multiple sclerosis [40] and diabetic nephropathy [41]. Similarly, *HSP90AA2* polymorphisms are reported to be associated with susceptibility to systemic lupus erythematosus [42]. However, we note that the mechanism that underpins the heterogenous advantage in relation to EHS is not fully understood. Similarly, the functional effect of the AG polymorphism on the expression of HSPA1B in heat tolerance and EHS is unknown.

Furthermore, genetic studies on heat illnesses in humans and animals have largely focused on gene expressions, such as the expressions of HSPA1B during heat stress or illness. In animal studies, HSPA1B has been reported to be expressed at very high levels in heat-stressed rats [43]. The HSPA1B expression was proportional to the severity of heat stress in baboons [44]. Among individuals with a history of EHI, HSPA1B was found to be upregulated [23]. Given the limited evidence, a more comprehensive understanding of the relationship between EHS and the HSP will require data from a larger sample of military personnel.

The fact that there were no differences between the genotypes at all HSP SNP loci in relation to heat tolerance status irrespective of EHS history is a pointer to the fact that heat tolerance is multifactorial and may be driven by other predisposing physiological and environmental factors [17,31]. Similar findings were also reported in studies for other health conditions. For example, evidence suggests that there is no significant association between *HSPA1B* SNP and exertional rhabdomyolysis [45]. Similarly, *HSPA1B* polymorphisms were not associated with elevated creatine kinase levels among military personnel following exertion [46]. The findings from the current study suggest that more research is warranted to explore the SNP associated with heat tolerance.

### 4.1. Strengths and Limitations

This study is the first to investigate genetic polymorphisms associated with heat intolerance and EHS in military populations. However, interpretation of the findings needs to be cautiously applied to other settings due to some inherent limitations of the study. The recruitment of control individuals from the ADF was not feasible due to ethical issues related to heat intolerance. For this study, only ADF members with a history of EHS who were referred and volunteered to participate in the HTT were recruited. Nevertheless, volunteers for the control group were chosen from the general population and matched to the patients in terms of age, gender, and aerobic fitness. The sample population of participants with EHS history was small. A larger sample size may provide robustness and wider applicability to the current findings.

### 4.2. Implications for Policy and Future Studies

Our study identified a significant difference in the genotypes of heat tolerant ADF participants with a history of EHS in comparison to their heat intolerant counterparts at the *HSPA1B* SNP g.31829044 locus. Given that the AG genotype is considered protective, the presence of this genotype may indicate some level of protection against EHI. However, the underlying physiological mechanisms how the genotype impose effects on EHS is unclear. Thus, it is unknown if the identified AG genotype in heat tolerant ADF members is indicative of recovery from EHS or cytoprotection against heat stress. Therefore, performing more in vitro and in vivo research on the association between polymorphisms of *HSPA1B* gene and EHS and heat tolerance is necessary, which could contribute to the return to duty process. This may also point directions toward other potential risk factors that could have caused the initial EHS episode.

Additionally, it is important for future studies to investigate the functional significance of the *HSPA1B* SNP and its correlations with mRNA gene expression. Apart from *HSPA1B*, which is considered a universal stress induced HSP [43], it would be beneficial to consider other HSP and their subclasses. Other genetic factors that are beyond the scope of this study may be involved in the regulation of heat tolerance. Therefore, large-scale studies are warranted to investigate the genetic polymorphisms that may be associated with heat tolerance and further functional analyses of the *HSPA1B* SNP g.31829044. Furthermore, given that genetic traits are passed from generation to generation, it would be beneficial for future studies to consider family-based hereditary pattern association studies on heat intolerance. The results in the present study are useful because EHI remains a significant threat among military personnel and may precede or accompany heat intolerance [12,47].

## 5. Conclusions

While the genetic basis for heat intolerance was not established among the participants in this study, the findings indicate an association between the *HSPA1B* genetic variation at the g.31829044 locus and heat tolerance among ADF participants with a history of EHS. However, many other mechanisms could have contributed to the above-mentioned association between the *HSPA1B* genetic variation and heat tolerance. Further research with larger participant numbers is needed, and the functional role of *HSPA1B* SNP at the g.31829044 locus in heat tolerance requires further investigation.

## Figures and Tables

**Table 1:** Study population characteristics by heat tolerance status.

Characteristics	Heat Tolerant	Heat Intolerant	*p*-Value
Age	31 (15)	27 (16)	0.252
**Gender**			
Male	17 (63.0%)	17 (81.0%)	0.174
Female	10 (37.0%)	4 (19.0%)	
**EHS history**			
Previous history of EHS	6 (22.2%)	8 (38.1%)	0.230
No history of EHS	21 (77.8%)	13 (61.9%)	

**Table 2:** HSPA1B and HSP90AA2 SNP genotypes by heat tolerance status (proportions %).

Gene	SNP Locus	Genotype	Heat Tolerant	Heat Intolerant	*p*-Value
*HSPA1B* ^a^	g.31829044G>A	AA	8 (30.8%)	9 (45.0%)	0.489
		AG	15 (57.7%)	8 (40.0%)	
		GG	3 (11.5%)	3 (15.0%)	
	g.31829851G>C	GG	7 (26.9%)	5 (25.0%)	0.690
		CG	14 (56.8%)	9 (45.0%)	
		CC	5 (19.2%)	6 (30.0%)	
*HSP90AA2* ^b^	g.27889377T>A	AA	19 (70.4%)	14 (70.0%)	0.978
		AT	8 (29.6%)	6 (30.0%)	
	g.27890100C>T	CC	19 (70.4%)	14 (70.0%)	0.978
		CT	8 (29.6%)	6 (30.0%)	
	g.27890332T>C	TT	19 (70.4%)	14 (70.0%)	0.978
		CT	8 (29.6%)	6 (30.0%)	

^a^ 2 missing observations; ^b^ 1 missing observation.

**Table 3:** Residual correlations between the HSP SNP, final core temperature and heart rate.

Gene	SNP Locus	Final Core Temp	Final HR
*HSPA1B*	g.31829044G>A	−0.05	−0.106
	g.31829851 G>C	0.072	0.118
*HSP90AA2*	g.27889377T>C	−0.041	0.063
	g.27890100C>T	−0.041	0.063
	g.27890332T>C	−0.041	0.063

**Table 4:** Genotype variation in HSPA1B and HSP90AA2 SNP by EHS history.

Gene	SNP Locus	Genotype	EHS History	No EHS History	*p*-Value
*HSPA1B* ^a^	g.31829044G>A	AA	4 (28.6%)	13 (40.6%)	0.736
		AG	8 (57.1%)	15 (46.9%)	
		GG	2 (14.3%)	4 (12.5%)	
	g.31829851G>C	GG	4 (28.6%)	8 (25.0%)	0.360
		CG	5 (35.7%)	18 (56.3%)	
		CC	5 (35.7%)	6 (18.8%)	
*HSP90AA2* ^b^	g.27889377T>A	AA	9 (64.3%)	24 (72.7%)	0.563
		AT	5 (35.7%)	9 (27.3%)	
	g.27890100C>T	CC	9 (64.3%)	24 (72.7%)	0.563
		CT	5 (35.7%)	9 (27.3%)	
	g.27890332T>C	TT	9 (64.3%)	24 (72.7%)	0.563
		CT	5 (35.7%)	9 (27.3%)	

^a^ 2 missing observations; ^b^ 1 missing observation.

**Table 5:** Stratified analysis of HSP SNP genotypes by EHS history and level of heat tolerance.

		EHS History					No History of EHS			
Gene	SNP	Genotype	Heat Tolerant n (%)	Heat Intolerant n (%)	*p*-Value	FDR *p*-Value	Heat Tolerant n (%)	Heat Intolerant n (%)	*p*-Value	FDR *p*-Value
*HSPA1B*	g.31829044G>A	AA	0 (0%)	4 (50%)	**0.024**	**0.048**	8 (40.0%)	5 (41.7%)	1	1
		AG	6 (100%)	2 (25%)			9 (45.5%)	6 (50.0%)		
		GG	0 (0%)	2 (25%)			3 (15.0%)	1 (8.3%)		
	g.31829851G>C	GG	1 (16.7%)	3 (37.5%)	0.201	0.402	6 (30.0%)	2 (16.7%)	0.703	0.703
		CG	4 (66.7%)	1 (12.5%)			10 (50.0%)	8 (66.7%)		
		CC	1 (16.7%)	4 (50.0%)			4 (20.0%)	2 (16.7%)		
*HSP90AA2*	g.27889377T>A	AA	4 (66.7%)	5 (62.5%)	0.872	0.872	15 (71.4%)	9 (75.0%)	0.825	0.872
		AT	2 (33.3%)	3 (37.5%)			6 (28.6%)	3 (25.0%)		
	g.27890100C>T	TT	4 (66.7%)	5 (62.5%)	0.872	0.872	15 (71.4%)	9 (75.0%)	0.825	0.872
		CT	2 (33.3%)	3 (37.5%)			6 (28.6%)	3 (25.0%)		
	g.27890332T>C	CC	4 (66.7%)	5 (62.5%)	0.872	0.872	15 (71.4%)	9 (75.0%)	0.825	0.872
		CT	2 (33.3%)	3 (37.5%)			6 (28.6%)	3 (25.0%)		

## Data Availability

All data generated or analysed during this study are included in this published article. The data for this study have been deposited at the James Cook University data repository at the following URL: https://research.jcu.edu.au/data/published/abd6de00799b11ecae4bb7cd799d3a4a/ accessed on 20 January 2022.

## Supplementary Materials

The following supporting information can be downloaded at: https://www.mdpi.com/article/10.3390/biom12101465/s1, Table S1: Primer sequences for polymerase chain reaction assays; Table S2: HSP SNP genotypes and major allele frequencies.
