# Supplementary material for: Next Generation Sequencing of Genotype Variants and Genetic Association between Heat Shock Proteins HSPA1B Single Nucleotide Polymorphism at the g.31829044 Locus and Heat Tolerance: A Pilot Quasi-Experimental Study
Source: Biomolecules. 2022 Oct 12;12(10):1465. doi: 10.3390/biom12101465 (PMC9599234; doi:10.3390/biom12101465)
Supplement: Supplementary file 1 [file biomolecules-12-01465-s001.zip › biomolecules-1823625-supplementary.pdf]

biomolecules

Table S1. Primer sequences for polymerase chain reaction assays.

Gene		Sequence	Length (bp)	T_a (°C)	Fragment length (bp)
HSPA1B	Forward	GAG GGT CCG CTT CGT CTT TCG	21	60°C	2517
	Reverse	TGT AGT GTT TTC GCC AAG CAA AAA	24		
HSP90AA2	Forward	ACC GGG TCT GAG TTA TTT TTA AAC A	25	60°C	2922
	Reverse	AGG AGG AGG TTG AGA CAT TCG C	22		
DNAJA1	Forward	CTT TCC AGA ACG CTC GGT GAG A	22	60°C	14635
	Reverse	GCG AAA GTC AAA TGG TTT AAA GTA A	25		

Table S2. HSP SNP genotypes and major allele frequencies.

Gene	SNP locus	Genotype	Major allele frequency (%)
HSPA1B	g.31829044G>A	AG	62
	g.31829851G>C	CG	51
HSP90AA2	g.27889377T>A	AA	85
	g.27890100C>T	TT	85
	g.27890332T>C	CC	85
